# Sexual dysfunction and its determinants in Moroccan women with rheumatoid arthritis

**DOI:** 10.11604/pamj.2016.24.16.9081

**Published:** 2016-05-06

**Authors:** Dina Khnaba, Samira Rostom, Racha Lahlou, Rachid Bahiri, Redouane Abouqal, Najia Hajjaj-Hassouni

**Affiliations:** 1 Mohammed V^th^ University, Ibn Sina Universitary Hospitals, El Ayachi Hospital, Department of Rheumatology, Rabat-Salé, Maroc; 2 Laboratory of Biostatistical, Clinical and Epidemiological Research, Faculty of Medicine and Pharmacy, Mohammed V^th^ University, Rabat, Maroc

**Keywords:** Rheumatoid arthritis, sexual dysfunction, pain, depression

## Abstract

**Introduction:**

To assess the prevalence of sexual dysfunction in married women with rheumatoid arthritis (RA) and compare it with a control group and to determine its association with clinical and disease activity factors.

**Methods:**

We conducted a cross-sectional study including sixty married women with a confirmed diagnosis of Rheumatoid Arthritis according to the American College of Rheumatology (ACR)/ European League against Rheumatism (EULAR) 2010 Criteria, aged 18 or over and having sexual activity. Our controls were healthy volunteers women matched for age. Clinical and sociodemographic characteristics were collected. Sexual function was assessed by a self-reported questionnaire the index of female sexual function (FSFI). Sociodemographic and disease activity profiles were compared between those who had and did not have sexual dysfunction.

**Results:**

The prevalence of female sexual dysfunction in women with rheumatoid arthritis attending El Ayachi hospital was 71.9%, it was 54% in controls. There was a significant difference in the total FSFI score between patients 18.29±9.09 and controls 23.05±7.91 (p=0.016). We found a statistically significant difference between the two groups in almost all dimensions of sexual function (desire, arousal, orgasm, satisfaction), except for pain and lubrication. In multivariate analysis, pain assessed by visual analogue scale (VAS) and depression assessed by hospital anxiety and depression score (HAD) were the independent determinants of sexual dysfunction.

**Conclusion:**

Our study suggests that sexual dysfunction is more common among patients with RA compared to controls. These dysfunctions were related to desire, arousal, orgasm and satisfaction. Pain and depression appear to be the most important predictors of sexual dysfunction.

## Introduction

Rheumatoid arthritis (RA) is a systemic chronic inflammatory disease leading to significant morbidity and disability [1, 2]. Fatigue, pain and decreased joint mobility caused by this rheumatic disorder often influence sexual health of patients [3]. RA can affect sexual function due to many reasons: Pain, stiffness, physical restrictions and fatigue are the major complaints of these patients. They can also have problems with body image and self esteem. Their medications can also cause sexual problems. Studies on the subject are limited and show divergent results [4]. Sexual health is an important part of human wellbeing; it has been defined by the World Health Organization (WHO) as a state of physical, mental, emotional and social well-being in relation to sexuality [5]. Sexual function should not be excluded from the monitoring of physical and mental health in our daily practice. The definition of female sexual dysfunction (FSD) used in clinical research was not uniform before the year 2000, where The International Consensus Development Conference on Female Sexual Dysfunction defined female sexual dysfunction as a persistent or recurring decrease in sexual arousal, dyspareunia and difficulty or inability to achieve an orgasm and which leads to personal distress and relationship difficulties [6]. There are several methods for assessing sexual function; the most used on chronic diseases is the FSFI (Female Sexual Function Index) which is validated in different countries. The FSFI is a self administered questionnaire developed by Rosen in 2000 for assessing FSD. It consists of 19 item multidimensional self report instruments for assessing the key dimensions of sexual function such as: desire, arousal, lubrication, orgasm, satisfaction and pain during the last four weeks. This questionnaire is easy to administer and is able to differentiate between women with and without sexual dysfunction; it has a good discriminative validity, internal consistency and test–retest reliability [7]. In our Arabic and Islamic culture, discussion about sexuality may be perceived as a taboo subject, which may explain the lack of studies in this area [8]. In Morocco, there has not been any study about sexual dysfunction among RA women. This is the first study looking at the frequency of FSD in an RA population using the Moroccan Version of FSFI and exploring the relationship between female sexual dysfunction, disease activity and socio cultural factors [9].

## Methods

### Patients

This was a cross sectional, case control study including women with a confirmed diagnosis of Rheumatoid Arthritis, aged 18 or over and attending El Ayachi University Hospital. The American College of Rheumatology (ACR)/ European League against Rheumatism (EULAR) 2010 diagnosis criteria was used in this study. All the patients were married and had a sexual activity within the last 4 weeks prior to the time of data collection. Patients were selected based on the inclusion and exclusion criteria listed below. A written informed consent for participation in the study was obtained from participants. We approached 80 patients but we excluded 20.

### Inclusion criteria were

Female patients aged 18 years old or over, diagnosed with RA according to the ACR/EULAR 2010 criteria and married in a heterosexual relationship for the last 6 months.

### Exclusion criteria were

Patients known to have other chronic disease, psychiatric illness or taking medication that may impair sexual function. Control group were healthy married women volunteers matched for age. The difficulties of writing and learn were an exclusion criteria.

### Measurement tools: primary outcome measure

#### Female sexual dysfunction (FSD): assessed by Female Sexual Function Index (FSFI)

The FSFI is a 19-item multidimensional self-report questionnaire assessing sexual dysfunction in the domains of satisfaction, lubrication, desire, arousal, orgasm and pain during the previous four weeks. The score normally varies between 1.2 and 36. [7]. The definition of sexual dysfunction was considered by FSFI as a score less than or equal to 26.55 [10, 11]. We used the Moroccan version of the FSFI which is being validated [9].

### Secondary outcome measure

#### Disease activity

Rheumatoid Arthritis activity was assessed by DAS 28 ESR score (Disease Activity score erythrocyte sedimentation rate). It is calculated using a simple formula that measures tenderness and swollen joints (28 selected joints), visual analogue scale for patients own perception (global health assessment) and ESR. A DAS28 score less than 2.6 corresponds to remission, between 2.6 and 3.2 it corresponds to low disease activity, between 3.2 et 5.1 it's a moderate activity and more than 5.1 corresponds to a high activity [12].

#### Functional status

It was evaluated using the Health Assessment Questionnaire (HAQ). The questionnaire consists of eight sections: dressing, arising, eating, walking, personal hygiene, grip, reach and social activities. There are 2 or 3 questions for each section. It explores the functional impact of several rheumatic disorders including RA; it correlates significantly with disease activity and severity parameters. For our local environment, the Moroccan dialect version of HAQ has been validated with strong sensitivity, and is valid for assessing functional status of RA patients in the Moroccan cultural context [13].

### Quality of life Qol: Short Form36 (SF36)

The quality of life QoL was assessed with SF-36 questionnaire, which has been previously validated in the Moroccan population [14]. It's a 36 item questionnaire measuring quality of life (Qol) across eight domains: physical function, physical role, body pain, general health, vitality, social function, emotional role and mental health. The scores of the eight domains varies between 0 and 100. High scores correspond to less limitations or distress in the different dimensions [14].

### Psychological status: Hospital anxiety and depression scale HAD

It is a questionnaire commonly used to determine the level of anxiety and depression that a patient is experiencing. It is a fourteen item scale, where seven questions are related to anxiety and seven questions are related to depression Each item is scored from 0 to 3, a person can score between 0 and 21 for either anxiety or depression. We used the validate Arabic version to detect patients with anxiety or depression [15].

### Study protocol

After consent was received, patients answered a sociodemographic questionnaire, including age, marital status, educational level and professional activity. Disease-specific variables such as date of diagnosis of RA, disease duration, history of medication and physical examination were recorded. The Moroccan version of FSFI was self-administered by the patients in a private room to allow privacy. Patients were assessed using the DAS28, HAQ, the Visual Analogue Scale for Pain and fatigue (VAS), SF36 and HAD scale. All patients had routine blood investigations: ESR, C-reactive protein (CRP) rheumatoid factor RF and anti-citrulinated protein antibodies ACPA.

### Statistical analysis

Data analysis was performed using SPSS for windows version 18. Data was expressed as mean ± SD (standard deviation) (for normally distributed data) or median (25th, 75th centiles) (for non-normally distributed data). We divided our patients in 2 groups F+(with sexual dysfunction) and F- (without sexual dysfunction) and we analyzed the variables between these two groups using the independent sample t test (for normally distributed data), chi square (for qualitative data) and the Man -Whitney U-test (for Non-parametric data). We correlated the FSFI score and its domains to the different parameters of the patients and the disease using the Spearman test. Multivariate analysis was done for data found to be significant in bivariate analysis. Multiple logistic regressions were used to analyze predictors for FSD. Factors significant (P < 0.05) in unadjusted analyses were included in a logistic regression model to determine factors independently associated with sexual impact, with results presented as odds ratios (ORs) with 95% CI.

## Results

### Characteristics of patients with RA

Sixty patients who attended El Ayachi hospital were screened, the mean of the age of women with RA recruited was 45.18±8.76 years, forty four (73.3%) had a primary educational level and fifty-three (88.3%) had no professional activity. All patients were married, and eighteen of them (30%) were at menopause.

### Disease characteristics

All patients had established RA, twenty four (40%) of them were seropositive. The median of disease duration was 68(37.5-126.6) months. The mean of DAS28 ESR was 4.12±1.70 and the median of HAQ was 1(0.5-1.3).

### Sexual function

All our patients were assessed using the Moroccan version of FSFI. The mean of the score of FSFI among our patients was 18.3±9.1, forty six (71.9%) of them had a sexual dysfunction. The median of the different domains of FSFI were respectively: Desire 1.5(1.2-3); arousal 2.4(1.2-3.6); lubrification 3.15(1.2-3.6); orgasm 3(1.2-4.4); satisfaction 3.6(2.4-5.1); pain 5.8(2.17-6). Other characteristics of patients and disease are represented in Table 1 and Table 2.

**Table 1:** Sociodemographic and disease characteristics of patients with rheumatoid arthritis

Variable	Patients (N = 60)	
Age (years) 3	45.18±8.76	
Professional activity^1^	Without professional activity	53 (88.3%)
	With professional activity	7 (11.7%)
Educational level^1^	Primary	44 (73.3%)
	Secondary	14 (23.3%)
	Tertiary	2 (3.3%)
Menopause^1^	18 (30%)	
Disease duration (month)^2^	68 (37.5-126.6)	
Corticosteroid intake^1^	Yes	58(96.7%)
	No	2 (3.3%)
Cumulative dose of steroids (mg)^2^	9672.5 (3102.2- 20683.33)	
RA treatment^1^	Methotrexate	50 (83.3%)
	Salazopyrin	25(41.7%)
	Leflunomid	3(5%)
	Biological DMARDS	22(36.7%)
HAD anxiety^3^	9.58±4.25	
HAD Depression^3^	10.73±2.84	
Total score of HAD^3^	20.13±6.20	
SF36 Domains	Physical function^3^	47.75±21.60
	Limitation due to physical health^2^	12.74(0-42)
	Pain^3^	41.92±21.77
	General health^3^	40.61±16.08
	Energy^2^	45 (25-50)
	Social function^3^	45.58±19.16
	Limitation due to emotional problems^2^	4.02(0-21.26)
	emotional well-being^2^	40 (29-52)

RA: rheumatoid arthritis; .had: hospital anxiety and depression score; SF36: short form 36.

1 Number and percentage

2 Median and percentile

3 Mean and standard deviation.

**Table 2:** Disease characteristics and sexual function of patients

Variables	Patients (N = 60)	
VAS pain^3^	47.83±20.42	
VAS fatigue 3	45.33±20.62	
Number of tender joint^2^	5 (2.25-10)	
Number of swollen joint^2^	1.3 (0.16-4.28)	
ESR^2^	19 (9-34.75)	
CRP^2^	5.48 (4.55-15)	
DAS28ESR^3^	4.12±1.70	
RF^2^	29 (48.3%)	
ACPA^2^	24 (40%)	
HAQ^2^	1 (0.5-1.3)	
FSFI Domains^2^	Domain1 Desire	1.5(1.2-35)
	Domain2 arousal	2.4(1.2-3.6)
	Domain3 lubrication	3.15(1.2-3.6)
	Domain4 orgasm	3(1.2-4.4)
	Domain5 satisfaction	3.6(2.4-5.1)
	Domain6 pain	5.8(2.17-6)
Score of FSFI^3^	18.3±9.1	
Presence of sexual dysfunction^1^	Patients with sexual dysfunction (F+)	46 (71.9%)

VAS: visual analogue scale; ESR: erythrocyte sedimentation rate; CRP: C reactive protein; DAS28:Disease Activity score; RF: rheumatoid factor; ACPA: anti-citrulinated protein antibodies; haq: health assessment questionnaire; FSFI: female sexual function index

1 Number and percentage

2 Median and percentile

3 Mean and standard deviation

### Comparison between women with RA and controls

After comparing our sixty patients and the thirty-one healthy volunteer controls for all domains of FSFI, we found a statistically significant difference between the two groups in almost all dimensions of sexual function (desire(p <0.0001), arousal(p =0.004), orgasm(p =0.004), satisfaction(p =0.004)), except for pain (p =0.20)and lubrication (p =0.13).

### Comparison between RA women with and without sexual dysfunction

After Comparison between RA women with (F+) and without (F-) sexual dysfunction, we found as shown in Table 3 that: there was no significant difference between patients with and without sexual dysfunction in term of age (mean of age of patients F+ and F-were respectively 45.54±8.89 years and 44±8.53 with P = 0.56). However they differed in term of educational level (p =0.033).

**Table 3:** Comparison between patients with (F+) and without (F-) sexual dysfunction

Variables	Patients with sexual dysfunction	Patients without sexual dysfunction	*P*
Age^3^	45±8.89	44±8.53	0.56
Disease duration(month)^2^	72(48-132)	27(12-111)	0.044
Patient with professional activity^1^	3(42.9%)	4(57.1%)	0.026
Educational level^1^			
Primary	35(76.1%)	9(64.3%)	
Secondary	11(23.9%)	3(21.4%)	0.033
Tertiary	0(0%)	2(14.3%)	
Cumulative dose of steroids^2^	12600(5475-25550)	36.50(1825-3650)	0.015
VAS pain^3^	51.95±18.33	34.28±21.73	0.004
VAS fatigue^3^	47.60±20.11	40.71±24	0.12
ESR^2^	24(8.75-45)	12(8.75-12)	0.19
CRP^2^	6(5-22.5)	5(4-6)	0.079
DAS28ESR^3^	4.27±1.77	3.62±1.36	0.21
HAQ^2^	1(0.5-1.3)	0.8(0.3-1)	0.081
HAD ANXIETY^3^	10.28±4.21	7.28±3.6	0.02
HAD DEPRESSION^3^	11.41±2.5	8.5±2.79	<0.00001
Total score of HAD^3^	21.17±6.03	16.92±5.78	0.03
SF36 physical score^2^	31.87(19.42-39.47)	45.4(32.34-61.83)	0.081
SF36 mental score^2^	30.66(20.06-36.5)	93.93(28.46-49.33)	0.006

VAS: visual analogue scale; ESR: erythrocyte sedimentation rate; CRP: C reactive protein; DAS28 Disease Activity score; HAQ: health assessment questionnaire.had: hospital anxiety and depression score; SF36: short form 36

1 Number and percentage

2 Median and percentile

3 Mean and standard deviation

Patients with sexual dysfunction had significantly longer duration of disease (p = 0.044) and a higher cumulative dose of corticosteroid (p=0.015) compared to those without sexual dysfunction. Patients with sexual dysfunction reported higher pain expressed by VAS than patients without sexual dysfunction (p=0.004). Disease activity measured using DAS28ESR, ESR and CRP level did not differ between those with and without sexual dysfunction.

Both patients with (F+) and without (F-) sexual dysfunction had similar HAQ scores for assessing disability. There was no difference between patients with and without sexual dysfunction in disability grading (p=0.081). They differed in term of mental status assessed by both HAD (p=0.03) and SF36 score (p=0.006) (Table 3).

### Predictors of FSD

In univariate analysis, the determinant factors of sexual dysfunction were pain assessed by VAS, anxiety and depression assessed by HAD and QOL assessed by physical and mental score of SF36 as shown in Table 4 and Table 5. In multivariate analysis pain assessed by VAS and depression assessed by HAD were the independent determinants of sexual dysfunction with respectively (Expβ=0.923; CI 95% between 0.866 and 0.998; p=0.043 and Expβ=0.698; CI 95% between 0.480 and 1.016; p=0.05).

**Table 4:** Sociodemographic and disease activity determinants of sexual dysfunction in univariate and multivariate analysis

Variable	Univariate analyis			Multivariate analysis		
	Exp β	CI 95%	*p*	Exp β	CI 95%	*P*
Age	0.98	0.919-1.048	0.562	-	-	-
Menopause	0.26	0.051-1.342	0.108	-	-	-
Educational level	1.523	0.826-2.810	0.178	-	-	-
Disease duration	0.991	0.979-1.002	0.107	-	-	-
VAS pain	0.954	0.921-0.987	0.008	0.929	0.866-0.998	0.043
VAS fatigue	0.976	0.946-1,007	0.125	-	-	-
Tender joint	0.915	0.8-1.047	0.198	-	-	-
ESR	0.971	0.935-1.009	0.131	-	-	-
DAS28ESR	0.788	0.539-1.150	0.216	-	-	-

Exp: exponential; CI: confidence interval

VAS: visual analogue scale; ESR: erythrocyte sedimentation rate; DAS28 Disease Activity score

**Table 5:** Psychological and Qol determinants of sexual dysfunction

variable	Univariate analysis			Multivariate analysis		
	Exp β	CI 95%	*p*	Exp β	CI 95%	*P*
HAD anxiety	0.828	0.700-0.978	0.026	-	-	-
HAD depression	0.621	0.456-0.846	0.003	0.698	0.48-1.016	0.05
SF36Physical score	1.035	1.003-1.068	0.029	-	-	-
SF36 Mental score	1.063	1.009-1.119	0.021	-	-	-

Exp: exponential; CI: confidence interval

HAD: hospital anxiety and depression score;SF36:short form 36

### Correlation between FSFI domains and disease activity, functional and psychological status

After examining each FSFI domain score, Age had a negative significant correlation with almost all domains of FSFI (Table 6). We found also that VAS pain had a significant negative correlation with excitation (Spearman's rho = -0.291, P =0.024), satisfaction (Spearman's rho = -0.366, P = 0.004) and pain (Spearman's rho = -0.256, P = 0.049) (Table 6). DAS 28 ESR did not have any significant correlation with FSFI scores or its domains except for pain.

**Table 6:** Correlations between sociodemographic characteristics, disease activity, total score and domains of FSFI

Variables	Total score FSFI	Domaine1 desire	Domain2 Arousal	Domain3 lubrification	Domain4 orgasm	Domain5 satisfaction	Domain6 pain
Age	-0.425++	-0.532+	-0.313+	0.466++	-0.467++	-0.402	-0.285+
Disease duration	-0.258+	-0.164	0.151	-0.348++	-0.246	-0.212	-0.46
Corticosteroidcumulative dose	-0.339+	-0.181	-0.198	-0.364+	-0.289	-0.343+	-0.199
VAS pain	-0.347+	-0.227	-0.291+	-0.250	-0.130	-0.366++	-0.256+
VAS fatigue	-0.295+	-0.159	--0.246	-0.152	-0.183	-0.305+	-0.335++
NTJ	-0.250	-0.284+	-0.212	-0.131	-0.250	-0.279+	-0.229+
NSJ	-0.255+	-0.260	-0.195	-0.121	-0.190	-0.284+	-0.290+
ESR	-0.120	-0.107	-0.032	-0.072	-0.074	-0.068	-0.227
CRP	-0.361+	-0.358+	-0.427++	-0.399+	-0.359+	-0.393+	-0.155
DAS 28 ESR	-0.207	-0.170	-0.158	0.079	-0.144	-0.217	-0.269+
HAQ	-0.313+	-0.353+	-0.348++	-0.157	-0.198	-0.349++	0.021
HAD anxiety	-0.336+	-0.189	-0.30++	-0.210	-0.265++	-0.341++	-0.145
HAD depression	-0.521++	-0.284+	-0.459++	-0.444++	-0.408++	-0.530++	-0.276+
HAD total score	-0.290+	-0.077	-0.265	-0.149	-0.196	-0.324+	-0.254
SF36 PF	0.488++	0.448++	0.432++	0.406++	0.367++	0.494++	0.340+
LP	0.130	0.21	0.042	0.109	-0.005	0.122	0.128
B Pain	0.445++	0.355++	0.391++	0.389++	0.347++	0.441++	0.284+
GH	0.408++	0.268+	0.395++	0.345++	0.340++	0.459++	0.338++
Physical score	0.453++	0.380++	0.390++	0.382++	0.338++	0.465++	0.343++
VIT	0.525++	0.448++	0.488++	0.442++	0.414	0.558++	0.345++
SF	0.456++	0.456++	0.407++	0.361++	0.310+	0.40++	0.283+
LE	0.311+	0.262+	0.258+	0.215	0.195	0.313+	0.149
MH	0.466++	0.297+	0.427++	0.488++	0.349++	0.447++	0.417++
Mental score	0.511++	0.374++	0.456	0.485++	0.370++	0.477++	0.410

+P < 0.05, ++P < 0.001. Significant correlations are marked. Non-significant correlations are not marked

FSFI:female sexual function index;VAS: visual analogue scale; NTJ: Number of tender joint; .NSJ: number of swollen joint; ESR erythrocyte sedimentation rate; CRP: C reactive protein; DAS28 Disease Activity score; haq:health assessment questionaire.had:hospital anxiety and depression score; SF36:short form 36; PF: physical function, LP: physical role, BP: body pain, GH: general health, VIT: vitality, SF: social function, LE: emotional role; MH mental health

+p < 0.05 ++p < 0.001

We found a negative correlation between HAQ score and desire, arousal and satisfaction (with respectively Spearman's rho = -0.353, P = 0.007; Spearman's rho = -0.348, P = 0.008; Spearman's rho = -0.349, P = 0.002). HAD Depression scale had a significant negative correlation with FSFI total score and its domains. Almost all domains of SF 36 had significant positive correlation with FSFI domains (Table 6).

## Discussion

The aim of this study was to explore the prevalence of sexual dysfunction in women with RA and to evaluate its association with socio-demography, disease activity, functional and psychological status. Communication about sexuality is stills a taboo topic in our context. Indeed, many patients are reluctant to raise sexual activity as well as health professionals. These finding are consistent with a previous study in our country [16]. Despite our reserved culture, none of our patients refused to participate in this study.

The prevalence of FSD in our sample was respectively 71.9% of women with RA and 28.1% of healthy controls. The most affected dimensions of sexuality were: lower scores in the domains of desire, arousal, orgasm and satisfaction in women with RA than in controls. Our results are approximately similar to those found in the literature [17–20]. The percentage of RA patients experiencing sexual problems ranged in previous studies from 29 to 76% [17, 18]. To the best of our knowledge, there were few RA studies using FSFI as a measure of sexual dysfunction. Shahar and al assessed sexual function in women with RA using the FSFI score, they reported a lower prevalence of FSD (29.4%) [17]. Similarly, coskun and all reported that 68.75% of Turkish women with RA had a sexual dysfunction according to FSFI score versus 15% of healthy controls. Total score and all domains of FSFI, except of pain were lower in RA group versus control group [18]. In Frikha and colleague's study, 7 out of 10 women with RA had a sexual dysfunction assessed by FSFI score and all subscales of FSFI were affected [19]. Another Turkish study found a mean of total score of FSFI in patients with RA (22.6 ± 9.0) significantly lower than controls (34.6 ±8.3) [20]. In comparison, the prevalence of sexual dysfunction in rheumatic disease (including systemic lupus erythematosus; rheumatoid arthritis; systemic sclerosis; antiphospholipid antibody syndrome; and fibromyalgia) assessed using FSFI was 18.4% [11]. Ayden et al reported 54.2% of sexual dysfunction in patients with Fibromyalgia versus 15.8% in controls using the FSFI questionnaire [11]. Indeed, 36 to 70% of all patients with RA are experiencing reduced sexual health, and they think that the problem is directly or indirectly due to their rheumatoid disorder. Women report more joint difficulties than men especially during sexual activities [21]. Two large surveys conducted by patient's organizations in France evaluating respectively 7,700 and 1,200 patients with RA who completed the questionnaires show that 51% of patients reported an adverse impact of this disease on their Sexuality [22], 70% reported a negative impact of the disease on their sexuality and 72% reported never having discussed their sexuality issues with a health care professional [23]. On the other hand, sexual health of patients is still rarely evaluated during medical visits [11] even if 56% of RA patients think that sexual health is a very important subject [3]. A recent study shows that rheumatologists assessed sexual activity in only 12% of their patients. the main reason of this negligence were, in their opinion, lack of time, difficulty in communication on this subject, or thinking that the issue is not related to their field [24]. Other reasons explaining this lack of interest in the assessment of sexual dysfunction among RA patients is the embarrassment when discussing their sexual activity [25].

In the present study we tried to see if sexual function was affected by disease activity, disability, quality of life or psychological status. In univariate analysis, we found that pain assessed by VAS, psychological status assessed by HAD and Qol assessed by SF36 were the determinants of sexual dysfunction, but when entered simultaneously, only pain and depression seem to be the independent factors affecting the sexual function. These findings are consistent with previous studies: Yilmaz and al reported that pain, disease activity and depression were determined to have a negative effect on sexual functions of women with RA [20]. A literature review of the sexual health of women with RA found that pain, reduced joint mobility, fatigue, depression and body image alterations affected sexual health of these patients [26]. Abdel-Nasser and al found in their regression model that pain, age, and depression were the significant determinants for sexual dissatisfaction [27]. Indeed, sexual problems are common in patients with chronic pain and in those with symptoms of distress and depression [28]. In the same way, Helland and colleagues found that mental distress was one of the factors independently associated with perceived problems with sexual activity [29].

In the present study, it appears that the level of pain and depression play more significant roles compared to disease activity or other parameters in contributing to sexual dysfunction in women with RA. It should be noted that our study was a cross-sectional study, so it was impossible to determine the direction of any causality. Our sample was small and taken from a monocentric tertiary care setting, therefore, the patients may have more sever and active disease. So it's difficult to generalize the results. However, our strong points were that despite our reserved culture, we evaluated the sexual function of women with RA using a validated questionnaire.

## Conclusion

Our findings suggest that patients with RA, experience high levels of impairment of sexual function (71.9% of women with sexual dysfunction). Factors found to have an independent effect on sexual function were pain and depression. Hence, health providers should be aware that sexual impairment is an important consequence of RA; they must consider sexual function as a part of the assessment of disability or quality of life in patients and should guide them by providing information or referral to specialists when appropriate. In the other hand, a better control of pain and depression symptoms may lead us to a better management of sexual life of our patients.

### What is known about this topic

- Sexual dysfunction is very commun among women with Rheumatoid arthritis.
- There were few RA studies using FSFI as a measure of sexual dysfunction.
- Pain, age and depression seem to be the significant determinants for sexual dissatisfaction.

### What this study adds

- It is the first study looking at the frequency of FSD in an RA population using the Moroccan Version of FSFI and exploring the relationship between female sexual dysfunction, disease activity and socio cultural factors.
- Sexual dysfunction is frequent among Moroccan married women with RA.
- The most affected dimensions of sexuality in our population were: desire, arousal, orgasm and satisfaction.
